# Retraction: Androgen receptor transactivates KSHV noncoding RNA PAN to promote lytic replication–mediated oncogenesis: A mechanism of sex disparity in KS

**DOI:** 10.1371/journal.ppat.1014281

**Published:** 2026-06-04

**Authors:** The PLOS Pathogens Editors

After this article [1] was published, the *PLOS Pathogens* Editors identified concerns about compromised peer review, statistical analyses, ethics approvals, and with Figs 1, 5 and 8. Specifically,

- In the Fig 8C + Doxycycline panel, the x20 image for AR-pCDH-sf3 + siPAN appears to be derived from the x10 image for pCDH-sf3 + siCtrl, and overlap with the x20 image for pCDH-sf3 + siCtrl.
- The a-actin panel in Fig 1E appears similar to lanes 1–9 of the a-actin panel in Fig 5E despite representing different conditions.
- In Fig 8A, the x20 siAR panel does not appear to be derived from the corresponding x20 panel.
- In the Fig 8C - Doxycycline panel, the x20 pCDH-sf3 + siCtrl and x20 AR-pCDH-sf3 + siPAN images do not appear to be derived from the corresponding x10 images.
- In Fig 1C, the x100 panel for 2# Non-KS angioma α-AR does not appear to be derived from the x40 panel.
- There appears to be vertical discontinuities between lanes 12 and 13 in both western blot panels of Fig 5E.
- The Materials and methods section states that data were analyzed using Student t test, but several figures in [1] report graphs for which this statistical test may not be appropriate.
- The Ethics statement in [1] states that approval was obtained from the Institutional Ethics Committee of the First Teaching Hospital of Xinjiang Medical University, but none of the authors of [1] are affiliated with this institution.

Regarding the overlap between the + Doxycycline panels of Fig 8C, and the anti-actin panels of Figs 1E and 5E, corresponding author, XW, stated that the + Doxycycline x20 AR-pCDH-sf3 + siPAN panel of Fig 8C, and the a-actin panel of Fig 1E are incorrect.

During post-publication discussions, corresponding author, XW, provided multiple updated versions of Figs 8C and 8D. Regarding the different versions of Figure 8C provided, the marked areas in some x10 panels did not appear to align with the corresponding x20 panels. Regarding the different versions of Figure 8D provided, they appear to contain different data compared to each other and to Figure 8D in [1]. In addition, the underlying data provided for Fig 8D in [1] and for the updated versions of Fig 8D appear to show discrepancies in values and numbers of samples. PLOS also received contradictory responses during post-publication discussions on whether there is an error in the analysis of integrated optical density reported in Fig 8D. The *PLOS Pathogens* Editors therefore have concerns with the reliability of the results presented in Fig 8.

Regarding the statistical analysis, different responses were received during post-publication discussions regarding the test used to analyze the data. The *PLOS Pathogens* Editors consider this concern unresolved given it is not clear if an appropriate test for graphs with two independent variables and multiple conditions was used. In addition, much of the quantitative underlying data provided in post-publication discussions only contains two samples, despite the Materials and methods section and some figures legends in [1] stating that experiments were performed at least in triplicate. Corresponding author, XW, stated that whilst at least three biological replicates were assessed under the same conditions, representative results from only two independent experiments are shown in [1]. The *PLOS Pathogens* Editors therefore have concerns about inaccurate sample size reporting and potential selective use of data in [1].

Regarding the Ethics statement, corresponding author, XW, stated that they were previously affiliated with Xinjiang Medical University, however, the ethics approval document provided in post-publication discussions does not list any authors of [1], and there appears to be a discrepancy in the project title and the title of [1].

In light of the above concerns which call into question the reliability of the results in [1], the *PLOS Pathogens* Editors retract this article.

KL agreed with the retraction and stands by the article’s findings. MD, JW, and XW did not agree with the retraction and stand by the article’s findings. RS, LY, LB, JS, HF and YZ either did not respond directly or could not be reached.
